# Public health research outputs from efficacy to dissemination: a bibliometric analysis

**DOI:** 10.1186/1471-2458-11-934

**Published:** 2011-12-15

**Authors:** Andrew J Milat, Adrian E Bauman, Sally Redman, Nada Curac

**Affiliations:** 1 Sax Institute, Sydney, Level 2, 10 Quay St Haymarket, NSW 2000, Australia; 2 School of Public Health, University of Sydney, Level 2, Medical Foundation Building, K25, NSW 2006 Sydney, Australia; 3 Current Address: Strategic Research and Development Branch, Centre for Health Advancement, NSW Ministry of Health, Locked Mail Bag 961, North Sydney, NSW 2059

## Abstract

**Background:**

More intervention research is needed, particularly 'real world' intervention replication and dissemination studies, to optimize improvements in health. This study assessed the proportion and type of published public health intervention research papers over time in physical activity and falls prevention, both important contributors to preventable morbidity and mortality.

**Methods:**

A keyword search was conducted, using Medline and PsycINFO to locate publications in 1988-1989, 1998-1999, and 2008-2009 for the two topic areas. In stage 1, a random sample of 1200 publications per time period for both topics were categorized as: non-public health, non-data-based public health, or data-based public health. In stage 2 data-based public health articles were further classified as measurement, descriptive, etiological or intervention research. Finally, intervention papers were categorized as: efficacy, intervention replication or dissemination studies. Inter-rater reliability of paper classification was 88%.

**Results:**

Descriptive studies were the most common data-based papers across all time periods (1988-89; 1998-1999;2008-2009) for both issues (physical activity: 47%; 54%; 65% and falls 75%; 64%; 63%), increasing significantly over time for physical activity. The proportion of intervention publications did not increase over time for physical activity comprising 23% across all time periods and fluctuated for falls across the time periods (10%; 21%; 17%). The proportion of intervention articles that were replication studies increased over the three time periods for physical activity (0%; 2%; 11%) and for falls (0%; 22%; 35%). Dissemination studies first appeared in the literature in 2008-2009, making up only 3% of physical activity and 7% of falls intervention studies.

**Conclusions:**

Intervention research studies remain only a modest proportion of all published studies in physical activity and falls prevention; the majority of the intervention studies, are efficacy studies although there is growing evidence of a move towards replication and dissemination studies, which may have greater potential for improving population health.

## Background

Research evidence can enhance public health policy and practice by assisting in the identification and definition of priorities, informing decisions on policy development and implementation and by evaluating the impact of policies and programs [1,2]. There is increasing recognition of the potential value of research evidence as one of the many factors considered by policy makers and practitioners [2,3]. Recent years have seen significant efforts by governments and health research funding agencies to facilitate closer links between policy makers and researchers and to target funding to increase the uptake of evidence into policy and practice [4,5].

**Figure 1:**
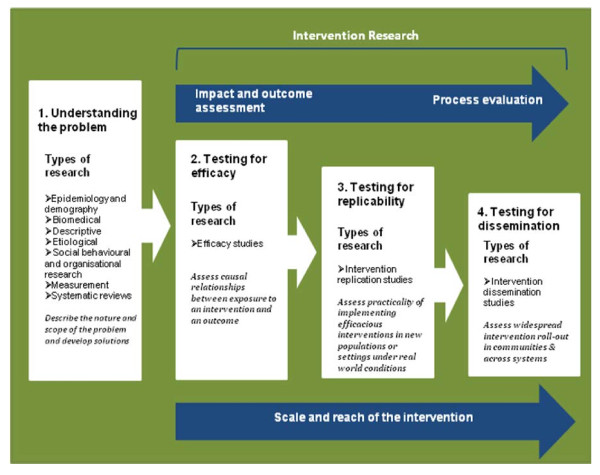
**Public health research progression model**. Adapted from: Nutbeam D and Bauman A: *Evaluation in a Nutshell - A Practical Guide to Evaluation of Health Promotion programs*. Sydney; McGraw Hill; 2006

Despite these efforts, the transfer of new knowledge from research into practice continues to be sub-optimal [6]. It has been argued that a contributing factor is the lack of research evidence that is optimally useful to policy makers, particularly the shortage of more generalizable policy and practice focused intervention research [7]. Figure 1 provides a conceptualization of research reflecting the different kinds of research that might be undertaken in each stage of development of an intervention, policy or program; this conceptualization is loosely based on the Nutbeam and Bauman (2006) Stages of Research and Evaluation model [8] (Figure 1), a literature review and input from intervention research experts.

Figure 1 describes public health research progression from descriptive studies undertaken to understand a problem, through to intervention dissemination studies testing widespread implementation and adoption of interventions. It is acknowledged that the progression described in the diagram represents an ideal and that intervention research development doesn't always progress in a linear fashion.

In this conceptualization, research that is targeted at understanding the problem includes measurement studies, descriptive studies exploring the frequency, patterns, correlates or predictors of a behavior or health issue and etiological studies, that is, epidemiological and other research studies that investigated a causal relationship between exposure to a risk factor and subsequent illness, disease or health outcome of public health significance.

Efficacy studies are those which evaluate the impact of an intervention (whether it does more good than harm among individuals in the target population) typically when delivered under optimal conditions (or in an ideal setting) [9]. Though there is a distinction between 'explanatory trials' which are implemented under highly controlled conditions and 'pragmatic trials' which are delivered in real world practice [10], using the Nutbeam and Bauman conceptualization both are considered efficacy research as they use random allocation of participants and their primary focus is on internal validity, establishing causal relationships between an intervention and an outcome [11]. They are also geared to focus on outcomes rather than process evaluation or contextual factors and as such may have poor generalizability [12].

Intervention replication studies are those which assess the practicality of implementing interventions with demonstrated efficacy, in new populations or settings under real world conditions [8]. They examine whether the core components of the original intervention are faithfully transported to the real-world setting (i.e. the degree of fidelity of the intervention with the original efficacy study), shifting the emphasis from outcomes to consider more closely how to identify the conditions for success. Intervention replication research also begins to assess the impact of the climate and culture in an organization or community on intervention effectiveness [8,12].

Finally, dissemination studies systematically study processes and factors that lead to widespread use of an evidence-based intervention across a target population or whole population. Its focus is to identify the best methods to enhance the widespread uptake and utilization of effective interventions [11,12]. Typically in dissemination research, the relative balance between outcome and process evaluation has moved further still, with the primary focus on measuring the process of change and assessing the reach of program dissemination across settings and target populations as the scale of intervention increases. This research involves a comprehensive assessment of impact of the climate and culture in an organization or community on intervention effectiveness to inform potential system adoption or widespread intervention roll-out in communities. As such this research can also measure program implementation in the context of quality management systems [8,13].

It is increasingly acknowledged that in order to maximize impact on health outcomes, intervention research must move beyond efficacy studies [14,15] to intervention replication and dissemination studies [8,16]. These study types implement interventions, ideally with demonstrated efficacy, under real life conditions and examine practical issues important to policy makers such as the extent to which a program can be adapted to meet variations in local need, circumstances and at what cost. These studies lead to widespread implementation and adoption of interventions into usual public health practice [8]. Without more intervention replication and in particular dissemination studies, the potential contribution of research to policy and practice is unlikely to be realized [15,17].

Recent efforts have been made to codify and quantify the types of research published in the peer-reviewed literature using variants of the typology shown in Figure 1[7]. Sanson-Fisher et al., (2008) found a consistent dearth of intervention research in the fields of smoking, alcohol and physical activity, constituting only 9% to 31% of publications, while descriptive research was the most commonly published research type across time (1987-88, 1997-98 and 2005-06) [7]. However, this study did not distinguish between the types of intervention research conducted.

In the present study, the issues of physical inactivity and injurious falls were examined as both make significant contributions to preventable morbidity and mortality in developed countries [18,19] and are targeted by public health and chronic disease prevention funding agencies [20]. Research in these areas can make major contributions to providing evidence to underpin important public health policy initiatives [21,22].

The aims of the study were to: a) determine the proportion of published literature for both health issues that were 'intervention research' and to further categorize 'intervention research' papers into three sub-categories of 'efficacy studies', 'intervention replication studies' and 'dissemination studies'; and b) to determine whether the relative proportion of 'intervention research' and it's sub-categories changed over three time periods.

This study differs from previous research in four important ways: first, it extends previous methods by classifying categorized 'intervention' papers into three new sub-categories to determine the proportion of intervention studies across the spectrum from efficacy to dissemination; second, the time period covered is longer than previous research; third, it assesses larger samples sizes of relevant papers, and finally, addresses a new health priority of falls prevention.

## Methods

### Data sources

A literature search was conducted using online databases Medline and PsycINFO to locate publications across three time periods: January 1988-December 1989, January 1998-December 1999, and January 2008-December 2009. Citations were located by key word searches and exploding medical subject headings (MeSH). A MeSH and key word pretesting process was undertaken prior to the final data collection using non study time periods in order to maximize the number of intervention studies identified by the search process and to refine the categorization system. A description of the final search process follows.

#### Physical activity

For Medline, the key word 'physical activity' and the MeSH terms, 'exercise' and 'physical fitness' were searched. For PsycINFO, the MeSH terms 'physical activity', 'exercise' and 'physical fitness' were searched. Abstracts were categorized as physical activity if they: examined physical activity as a primary research question and/or study outcome. While those that examined muscular movement without an intended health or performance benefit were excluded i.e. biomechanical studies describing physical impairment.

#### Falls Prevention in older people

For Medline, the key word 'falls' and the MeSH terms 'accidental falls', 'accident prevention', 'prevention', 'aged' were searched individually. For PsycINFO, the MeSH terms 'falls', 'accidental falls', 'accident prevention', 'prevention' and the key word 'aged' were searched. Followed by a combined MeSH and keyword search: accidental falls AND aged; accident prevention AND aged; falls AND aged; accidental falls AND prevention; falls AND prevention.

Falls were defined as an unexpected event in which a person comes to rest on the ground or floor or a lower level [23]. Falls prevention was considered a series of behavioral, medical, pharmacological and or environmental strategies employed to reduce the number of accidental falls suffered by older people [24]. Studies focusing on falls from heights or falls in populations other than older people were excluded. Falls prevention-related medical and pharmacological interventions were considered non-public health if they were not accompanied by some form of education, counseling, environmental and or health care delivery system change.

When the key word search generated a sample greater than 1200 papers for designated health issues the random number generation function in R Statistics [25] was used to randomly select 1200 records for inclusion in the study sample. The pilot testing coding of 300 papers per health area in a non-study time period suggested that a sample of 1200 would provide between 150-300 data-based public health articles per time period. It was estimated that this sample would be sufficient to estimate a difference in independent proportions of between 5-8% at the 95% confidence level. In stages 1 and 2 abstracts were independently coded by two of the authors (AJM, NC). In stage 3 full citations were used to classify intervention research papers independently by two authors and where different classifications emerged these were discussed amongst coders against the coding criteria and assigned a final categorization. Inter-rater reliability was assessed using approximately 50 cross over records for each time period yielding a total of n = 295 records for comparison. Inter-rater agreement was 88%.

In stage 1 relevant articles on topics of interest were first categorized into one of three categories: non-public health, non-data-based public health, or data-based public health [7]. For this study, research that described efforts to protect and promote health, and to prevent illness, injury and disability on a population or population subgroup level was considered 'public health'. Preventive non-pharmacological interventions conducted in clinical populations were classified as public health in this study, that is, it included studies focusing on primary, secondary and tertiary prevention [26]. Preventive interventions focusing on those suffering rare conditions with population prevalence rates of < 1% i.e. cystic fibrosis, were excluded.

#### Non-public health

These papers included laboratory and clinical research that explored the topic areas from physiological or basic science perspectives, for example, examination of the impact of physical activity on organ function or metabolism; or the effects of medications on bone density. When the effects of some form of education or counseling or system change were examined in addition to medication, the research was classified as 'public health'.

#### Non-data-based public health

These papers did not report new data. They included reviews, discussion papers, or commentaries, news, editorials, case reports, conference reports and descriptions of research projects or health programs. Meta-analyses were classified as non-data based public health in this study, as they are generally secondary analyses of already published primary data.

#### Data-based public health

These were original articles reporting new data, both quantitative and qualitative. Included papers examined: prevalence; health behaviors, and associated risk and protective factors have with health, illness, and other variables; determinants or correlates; knowledge and attitudes; and intervention research findings.

In stage 2 data-based public health research articles were further categorized into one of the following categories: measurement, descriptive, etiological or intervention. In Figure 1, measurement, descriptive and etiological studies would all be categorized as 'understanding the problem'.

#### Measurement

Papers developed or examined the qualities of a measurement instrument such as reliability, validity, or acceptability. Data collection methods included the use of questionnaires, interviews, physiological assessments, risk screening and observations. Papers that focused on both measurement and descriptive issues were coded as measurement research.

#### Descriptive

Papers explored the frequency, patterns, correlates or predictors of physical activity, or fall-related injury, or related variables such as knowledge, attitudes, healthcare practices, policy, or legislation. They included epidemiological studies examining frequency or patterns of risk factors and how these may be related to disease at a community or population level and other correlates research.

#### Etiological

Epidemiological and other research studies that investigated a causal relationship between exposure to a risk factor and subsequent illness, disease or health outcome of public health significance.

#### Intervention

Papers that tested the effectiveness of an intervention to modify preventive health-risk behaviors and/or the implementation of best practices by health care professionals. Intervention publications were defined by the research aims rather than the study design or type of intervention. Papers that focused on both descriptive and intervention issues were classified as intervention research. Intervention research was then further categorized.

While in third and final stage intervention papers were categorized as either efficacy studies, replications studies or dissemination studies as shown in Figure 1. These intervention types are described in the introduction and the specific assessment criteria used to categorize intervention studies is outlined in Table 1. In this study, explanatory trials and pragmatic trials not replicated in new populations or settings were categorized as efficacy studies.

**Table 1:** Assessment criteria for intervention research categorization

Type of Research	Study design	Intervention scale	Focus on outcome/impact measures	Focus on process evaluation	Internal validity	External validity (generaliz-ability)	Measures of system Adoption	Cost per participant	Population Reach
**1. Efficacy** **studies**	*RCT	*Limited to study subjects - small	High	Lower	Highest	Lower	Lower	*Higher	*Study subjects
	*Cluster RCT								

**2. Replication** **studies**	* RCTs	*Limited to study subjects (multiple sites) - moderate	High	Moderate	Moderate	Moderate	Moderate	*Moderate	*Study subjects in new target groups or settings
	*Cluster RCT								
	*Step wedge trial								
	*Quasi-experimental designs								

**3. Dissemination** **studies**	*Step wedge trial	*System level - large	Moderate	Highest	Lower	Higher	Higher	*Lower cost per participant.	*Broad population reach (Multiple populations and settings)
	*Quasi-experimental designs							*Measures of system adoption costs.	

After attempting to apply the above categorization a fourth classification emerged of low quality efficacy research. This latter category assessed the impact of small scale interventions using weak research designs including 'before and after studies' or measured impact of interventions using organizational quality assurance mechanisms. These studies were only identified in the falls prevention literature and as there were very few examples of these studies in the total sample (n = 10) they were collapsed into the efficacy research category.

#### Statistical analysis

Frequencies, chi square statistics and chi-square statistics for trend (*Χ*^2 ^_trend_) were calculated in winPEPI [27].

## Results

The total number of articles generated by the keyword searches for the two health issues (physical activity and falls prevention) varied from 318 to 24,540, across the designated time frames. Table 2 shows the number and percentage of publications generated by the searches; the relevant publications (from the randomly selected sample of 1200 where more than 1200 publications were identified); non-public health publications; and data-based and non-data-based public health publications for each issue over the three time periods. The number of publications relevant to the topic of physical activity remained stable overtime while those relevant to falls prevention increased substantially from 84 in 1988-89 to 659 in 2008-09.

**Table 2:** Number and percentage of publications classified as non-public health, data-based, or non-data-based public health

Issue	Time period	Publications generated by search	Publications relevant to topic^a^	Non-public health research^b^	Data-based public health research^b^	Non-data based public health research^b^
		**N**	**n**	**n %**	**n %**	**n %**

Physical activity	1988-1989^a^	5902	856	651 (76)	156 (18)	49 (6)
	
	1998-1999^a^	10147	854	539 (63)	251 (29)	64 (7)
	
	2008-2009^a^	24540	865	484 (56)	310 (36)	71 (8)

Falls prevention	1988-1989	318	84	32 (38)	40 (48)	12 (14)
	
	1998-1999	693	304	82 (27)	179 (59)	43 (14)
	
	2008-2009^a^	1891	659	191 (29)	363 (55)	105 (16)

^a ^From the sample of 1200 randomly selected publications

^b ^denominator is a random sample or total sample publications relevant to the topic for that year

Of the physical activity papers that were relevant to the topic, the proportion that were data-based public health increased significantly overtime (X^2^*trend *= 69.2, *p *= 0.001), from 18% in 1988-1989, 29% in 1998-1999 and 36% in 2008-2009. Of the falls papers that were relevant to the topic, the proportion identified as data-based publications increased initially from 48% in 1988-1989, to at 59% in 1998-1999 and stabilized at 55% in 2008-2009.

Of the data-based public health publications, the number and proportion of measurement, descriptive, etiological or intervention research articles for physical activity and falls at each time period are shown in Table 3.

**Table 3:** Number and percentage of data-based public health publications classified as measurement, descriptive, etiological or - intervention research

Issue	Time period		Measurement	Descriptive	Etiological	Intervention
		**N**	**n %**	**n %**	**n %**	**n %**

Physical activity	1988-1989	156	13 (8)	74 (47)	33 (21)	36 (23)
	
	1998-1999	251	18 (7)	136 (54)	39 (16)	58 (23)
	
	2008-2009	310	17 (5)	203 (65)	18 (6)	72 (23)

Falls prevention	1988-1989	40	3 (8)	30 (75)	3 (8)	4 (10)
	
	1998-1999	179	18 (10)	114 (64)	10 (6)	37 (21)
	
	2008-2009	363	67 (18)	228 (63)	8 (2)	60 (17)

Note: denominator is data-based publications for that year

For physical activity, there was a significant increase in the proportion of descriptive studies published over time (X^2^*trend *= 15.66, *p *= 0.001) from 47% in 1988-1989 to 65% in 2008-2009. The proportion of measurement articles dropped slightly over time constituting 8% of data-based public health papers in 1988-1989 and 5% in 2008-2009, while there was a significant decrease in the proportion of etiological studies published over time (X^2^*trend *= 23.54, *p *= 0.001). The proportion of intervention research publications remained stable over time staying at 23% of data-based publications across all time periods.

For falls prevention, the proportion of descriptive studies published progressively decreased over time from 75% in 1988-1989, 64% in 1998-1999 to 63% in 2008-2009. While, the proportion of measurement studies significantly increased over time from 8% in 1988-1989 to 18% in 2008-2009 (X^2^*trend *= 7.23, *p *= 0.007), etiological studies decreased significantly over time (X^2^*trend *= 7.53, *p *= 0.006) from 8% in 1988-1989 to 2% in 2008-2009. For falls intervention publications there was a notable but non-significant increase between 1988-1989 from 10% and to 21% in 1998-1999, followed by a modest drop to 17% in 2008-2009 (X^2^*trend *= 0.00, *p *= 0.96).

The number and percentage of intervention papers classified as either as efficacy, intervention replication and intervention dissemination studies for physical activity and falls prevention over each time period are shown in Table 4. Efficacy publications were the predominant intervention type published in the peer reviewed literature over time periods and topic areas. Due to the small sample sizes of intervention replication and dissemination studies, X^2^trends were not calculated. However, the proportion of intervention articles that were intervention replication studies increased over time for physical activity from zero in 1988-1989, 2% in 1998-1999 to 11% in 2008-2009 and for falls from zero in 1988-1989, 22% in 1998-1999 to 35% in 2008-2009. Despite these increases, dissemination studies only made up 3% and 7% of all intervention publications for physical activity and falls respectively and only appeared in the literature in 2008-2009.

**Table 4:** Number and percentage of intervention papers classified as efficacy, intervention replication and intervention dissemination studies

Issue	Time period		Efficacy	Replication	Dissemination
		**N**	**n %**	**n %**	**n %**

Physical activity	1988-1989	36	36 (100)	-	-

	1998-1999	60	59 (97)	1 (2)	-

	2008-2009	72	62 (86)	8 (11)	2 (3)

Falls Prevention	1988-1989	4	4 (100)	-	-

	1998-1999	37	29 (78)	8 (22)	-

	2008-2009	60	35 (58)	21 (35)	4 (7)

Note: denominator is intervention publications for that year

## Discussion

There was a significant increase in the proportion of data-based public health papers published over time for physical activity, but not for falls prevention. At all three time periods, publications in each topic were predominantly descriptive, with smaller numbers of intervention, measurement and etiological publications. The proportion of descriptive research studies increased significantly over time for physical activity, despite increasing policymaker interest in solutions generation [6]. While, descriptive research marginally declined for falls prevention perhaps reflecting differences in the research progression in the two fields.

Although recent statements by both governments and funding agencies indicate that more information is required about the impact of health policies and programs [4-6] the results of this study fail to demonstrate that more intervention research is occurring. Rates remained stable over the past three decades for physical activity (23%) and fluctuated for falls prevention (10% 1988-1989; 21% 1998-1999; 17% 2008-2009). The continued dominance of descriptive research in this as well as other earlier studies [7] is a matter for concern, with relatively few studies testing the effectiveness of interventions across the two topic areas. They highlight a consistent and ongoing under-representation of intervention research over time and across topic areas, suggesting that research development is not progressing toward its full potential in public health. In this review, the problem of sustained and even increased descriptive research applies to physical activity, an area in great need of generalizable evidence.

Descriptive research may be easier to conceptualize, complete and publish than intervention research [7]. In addition researchers may find it easier to attract research resources to undertake descriptive research, and such research is often less intrusive for participants, and may be more rapidly completed and published than intervention research [7]. This coupled with the fact that performance indicators of individual researchers and research groups usually incorporate the number of publications, grants, postgraduate research students, and the amount of competitive research funding as core performance metrics [28], there is ample incentive to focus on descriptive research over intervention research. Some argue that the system provides incentives for counting what can be measured rather than measuring what counts [29].

Efficacy studies made up the majority of intervention publications across health issues and time periods. Though efficacy studies remain important, these optimally designed studies are often developed using a level of research resources and individual assessment that are not easily reproduced under real world conditions. Once efficacy is established, the relative scientific comfort of clinical trials needs to give way to the greater uncertainty of working in larger populations and with organizations and communities to engage in more generalizable program and intervention delivery [17].

As Greenhalgh (2004) states: *'Context and "confounders" lie at the heart of the diffusion, dissemination, and implementation of complex innovations. They are not extraneous to the object of study; they are an integral part of it.' *[12] p. 615. Greenhalgh stresses that the 'context' must become a legitimate objective for scientific study and a legitimate influence on decision making. Intervention replication and dissemination studies provide policy makers and practitioners with context specific information on what needs to be done, by whom, to what standard and at what cost, all prerequisites for optimal adoption of intervention programs into policy or practice.

Notably, there has however been some progression in the types of intervention research published over time, with the proportion of replication studies increasing from zero in 1988-1989 to11% in 2008-2009 for physical activity and of particular note, increasing from zero in 1988-1989 to 35% in 2008-2009 for falls. This latter estimate indicates nearly half of published falls prevention interventions are now more generalizable tests of the replicability of efficacy studies into new and broader settings.

Though it is difficult to speculate as to the reason for the difference in research progression between the fields, a likely contributor is the nature of the falls prevention action. The fact that most falls prevention interventions use individual level outcomes as units of measurement, may make them more amenable to evaluation using randomized control trial designs, subsequently making the transition from efficacy to replication studies easier than it is for population-based physical activity interventions.

However, there remains a dearth of examples of progression to the next arguably more important dissemination phase in the literature. Though dissemination studies increased over time for both issues 3% and 7% in 2008-2009 for physical activity and falls respectively, they still remain a very small proportion of published intervention research, particularly for physical activity. Although rare, current dissemination studies suggest directions for the future, for example, Fortinsky et al. 2008 [30] examined the extent to which fall risk assessment and management practices for older patients were implemented in 19 Medicare-certified home health agencies (HHAs) in southern New England, United States of America. Our findings clearly demonstrate that such systematic study of processes and factors that lead to widespread use of evidence-based interventions remain the exception rather than the rule [31].

Though there has been some progress in the area of falls prevention, this study reinforces the need for active efforts by funding agencies, research community and governments alike to foster strategic progression of studies across the continuum from efficacy through to dissemination with the ultimate aim of impacting on policy and practice. Though there is increasing acknowledgement of the importance of research translation and funding intervention research [15], our findings suggest that significant cultural and system change are still required amongst funding decision makers and researchers alike.

The limitations of randomized controlled trial designs for population-level dissemination evidence needs to be better acknowledged by research publishers and funders [32]. Funding agencies and journals should be encouraged to support the generation and publication of more methodologically rigorous intervention replication and dissemination studies. Where appropriate journals should apply broader reviewer and reporting criteria that take into account guidance such as the Medical Research Council 'Developing and evaluating complex interventions'[33], Cochrane Collaboration 'Effective Practice and Organization of Care (EPOC) Criteria for reviewing study designs and data quality' [34] and Consolidated Standards of Reporting Trials (CONSORT) statement [10]. While funding agencies should also adopt these broader interpretations of research design commensurate with generating policy-relevant evidence [35], as well as funding more targeted dissemination research grant schemes.

Closer examination of other health areas may again reveal considerable diversity in the extent to which good-quality intervention studies are available for particular topics. This study highlights the importance of the investigation of research progression in other areas as a way of identifying evidence-generating research needs. Investigations of this kind are also needed to shed light on whether the translational research rhetoric matches reality.

The current study has built on bibliometric methods previously used in the published literature [7]. While there may be individual variation in coding, these differences are unlikely to change the trend data, proportion sampled or inter-topic differences. The literature search used in this study provided a snapshot of research outputs within three selected time periods; but as with previous work in this area [7] the use of only two major health literature databases may under-estimate the true number of published articles. However, this underestimation is likely to be non-differential, in that it would not change relationships in these trends in publication. Another potential limitation of this study is its reliance on MeSH search terms, which have been shown in some instances to identify fewer published studies [36]. Though this study predominantly used MeSH search terms, it also employed key words searches that were pretested prior to use to ensure higher capture rates for relevant intervention articles.

Though dissertations were included in this review, policy documents, conference abstracts, and grey literature published for government departments were not. Sanson-Fisher et al., (2008) argue that well-designed studies are likely to be published in peer-reviewed journals and that it is unlikely that rigorous intervention studies would be under-represented as a result [7]. Based on the lack of dissemination research found in this study, we surmise that either this research is not occurring or that it may be more likely to be published in the grey literature. As such, it is recommended that future assessments of intervention research outputs should attempt to include these types of literature despite the methodological challenges this may present.

## Conclusions

Despite recent efforts by policy makers and funders to increase intervention research outputs, there remains a need to increase the quantity and quality of such research, with a greater focus on the conduct and publication of intervention replication and particularly dissemination studies.

## Competing interests

The authors declare that they have no competing interests.

## Authors' contributions

AJM developed the concept for the paper, independently conducted the literature search, conducted the sample extraction, assessed and categorized papers, conducted the analyses and led manuscript production. AB was involved in the development of the concept, direction, drafting and editing of the manuscript. He also rechecked the abstracts for study inclusion and assisted with the data analysis. SR was involved in the direction and concept of the paper and commented on drafts of papers. NC assessed and categorized papers and commented on paper drafts. All authors have read and approved the manuscript.

## Pre-publication history

The pre-publication history for this paper can be accessed here:

http://www.biomedcentral.com/1471-2458/11/934/prepub

## References

[B1] Hanney SR Gonzalez-Block MA Buxton MJ Kogan M The utilisation of health research in policy-making: concepts, examples and methods of assessment Health Res Policy Syst 2003 1 2 29 10.1186/1478-4505-1-2 12646071 PMC151555

[B2] Mays N Pope C Popay J Systematically reviewing qualitative and quantitative evidence to inform management and policy-making in the health field J Health Ser Res Policy 2005 10 Supp 1 S6 S20 10.1258/1355819054308576 16053580

[B3] Bowen S Zwi A Sainsbury P What evidence informs government population health policy? Lessons from early childhood intervention policy in Australia NSW Public Health Bull 2005 16 180 184 10.1071/nb05050 16778918

[B4] National Health and Medical Research Council National Health and Medical Research Council (NHMRC) Strategic Plan 2007-2009 2007 Canberra: NHMRC

[B5] Canadian Institutes of Health Research. Health Research [CIHR] Roadmap: Creating innovative research for better health and health care CIHR Strategic Plan:2009/10 - 2013/14 2009 Ottawa, CIHR

[B6] Productivity Commission Strengthening Evidence-based policy in the Australian Federation, Volume 2: Background Paper 2010 Productivity Commission: Canberra

[B7] Sanson-Fisher RW Campbell EM Htun AT Bailey LJ Miller CJ We Are What We Do: Research Outputs of Public Health Am J Prev Med 2008 35 4 380 385 10.1016/j.amepre.2008.06.039 18687567

[B8] Nutbeam D Bauman A Evaluations in a Nutshell: a practical guide to the evaluation of health promotion programs 2006 Sydney: McGraw-Hill Companies

[B9] Rabin BA Brownson RC Haire-Joshu D Kreuter MW Weaver NL A glossary of dissemination and implementation research in health J Public Health Management Practice 2008 14 2 117 123 10.1097/01.PHH.0000311888.06252.bb 18287916

[B10] Zwarenstein M Treweek S Gagnier JJ Douglas G Altman DG Tunis S Haynes B Oxman AD Moher D for the CONSORT and Pragmatic Trials in Healthcare (Practihc) groups Improving the reporting of pragmatic trials: an extension of the CONSORT statement BMJ 2008 337 a2390 10.1136/bmj.a2390 19001484 PMC3266844

[B11] Higgins JPT, Green S Cochrane Handbook for Systematic Reviews of Interventions version 5.0.2 2009 Cochrane Collaboration

[B12] Greenhalgh T Robert G Macfarlane F Bate P Kyriakidou O Diffusion of innovations in service organizations: systematic review and recommendations Milbank Q 2004 82 581 629 10.1111/j.0887-378X.2004.00325.x 15595944 PMC2690184

[B13] Pajunen P Landgraf R Muylle F Neumann A Lindstrom J Schwarz PE Peltonen M Acosta T Adler M AlKerwi A Barengo N Barengo R Boavida JM Quality indicators for the prevention of type 2 diabetes in Europe - IMAGE Hor Metab Res 2010 42 Suppl 1 556 563 10.1055/s-0029-1240976 20391308

[B14] National Institute on Drug Abuse Blue Ribbon Task Force Report on Services Research 2004 United National Institutes of Health States Department of Human Services http://www.drugabuse.gov/about/organization/nacda/HSRReport.pdf

[B15] National Institutes of Health Dissemination and Implementation Research in Mental Health 2008 United States Department of Human Services http://www.cdc.gov/diabetes/projects/research.htm/Centers for Disease Control: Diabetes projects. *Translation research projects (TRIAD)*

[B16] Rubenstein LV Pugh J Strategies for promoting organizational and practice change by advancing implementation research Journal of General Internal Medicine 2006 21 Suppl 2 S58 S64 16637962 10.1111/j.1525-1497.2006.00364.x PMC2557137

[B17] National Institute of Health (NIH) Roadmap for medical research. Translational Research http://commonfund.nih.gov/clinicalresearch/overview-translational.aspx PMC3860449 23584747

[B18] American Geriatrics Society British Geriatrics Society American Academy of Orthopaedic Surgeons Panel on Falls Prevention Guideline for the prevention of falls in older persons Journal of the American Geriatrics Society 2001 49 664 672 10.1046/j.1532-5415.2001.49115.x 11380764

[B19] World Health Organisation (WHO) The global burden of disease: 2004 update 2008 Geneva: WHO

[B20] National Health and Medical Research Council National Health Priority Areas http://www.nhmrc.gov.au/grants/research-funding-statistics-and-data/funding-datasets/injury-related-issues

[B21] Shaw KA Gennat HC O'Rourke P Del Mar C Exercise for overweight or obesity Cochrane Database of Systematic Reviews 2006 4 Art. No.: CD003817 doi: 10.1002/14651858.CD003817.pub3 10.1002/14651858.CD003817.pub3 PMC9017288 17054187

[B22] Sherrington C Whitney JC Lord SR Herbert RD Cumming RG Close JCT Effective exercise for the prevention of falls: A systematic review and meta-analysis Journal of the American Geriatrics Society 2008 56 2234 2243 10.1111/j.1532-5415.2008.02014.x 19093923

[B23] Lord S Sherrington C Menz H Close J Falls in older people: risk factors and strategies for prevention 2008 Cambridge: Cambridge University Press

[B24] Fall Prevention Center of Excellence Basics of Fall Prevention http://www.stopfalls.org/aboutus/index.shtml

[B25] R Statistics http://www.r-project.org/

[B26] Katz J, Peberdy A, Douglas J Promoting Health: Knowledge and Practice 2000 Basingstoke (UK): Macmillan Press

[B27] Abramson JH WINPEPI (PEPI-for-Windows): computer programs for epidemiologists Epidemiologic Perspectives & Innovations 2004 1 6 10.1186/1742-5573-1-6 15606913 PMC544871

[B28] Taylor J The impact of performance indicators on the work of university academics: evidence from Australian universities Higher Education Quarterly 2008 55 42 61

[B29] Wells R Whitworth JA Assessing outcomes of health and medical research: do we measure what counts or count what we can measure? Aust New Zealand Health Policy 2007 4 14 10.1186/1743-8462-4-14 17597545 PMC1929109

[B30] Fortinsky RH Baker D Gottschalk M King M Trella P Tinetti ME Extent of implementation of evidence-based fall prevention practices for older patients in home health care Journal of the American Geriatrics Society 2008 56 737 743 10.1111/j.1532-5415.2007.01630.x 18284538

[B31] Milat AJ King L Bauman A Redman S Scaling up health promotion interventions: an emerging concept in implementation science Health Promotion J of Australia 2011 22 238 10.1071/he11238 22497071

[B32] Sanson-Fisher RW Bonevski B Green LW D'Este C Limitations of the Randomized Controlled Trial in Evaluating Population-Based Health Interventions American J Prev Med 2007 33 155 161 10.1016/j.amepre.2007.04.007 17673104

[B33] Medical Research Council Developing and evaluating complex interventions: new guidance http://www.sphsu.mrc.ac.uk/Complex_interventions_guidance.pdf

[B34] Cochrane Collaboration Effective Practice and Organisation of Care Review Group Data collection checklist http://epoc.cochrane.org/sites/epoc.cochrane.org/files/uploads/datacollectionchecklist.pdf

[B35] Glasgow RE Magid DJ Beck A Ritzwoller D Estabrooks PA Practical Clinical Trials for Translating Research to Practice Design and Measurement Recommendations Medical Care 2009 43 551 557 10.1097/01.mlr.0000163645.41407.09 15908849

[B36] Winchester DE Bavry AA Limitations of the MEDLINE database in constructing meta-analyses Ann Intern Med 2010 153 347 8 20820050 10.7326/0003-4819-153-5-201009070-00017

